# Reforms in medical education: lessons learnt from Kyrgyzstan

**DOI:** 10.1080/16549716.2021.1944480

**Published:** 2021-07-30

**Authors:** Gulzat Orozalieva, Louis Loutan, Aigul Azimova, Anne Baroffio, Olivia Heller, Bruno Lab, Altynai Mambetova, Damira Mambetalieva, Elvira Muratalieva, Mathieu Nendaz, Georges Savoldelli, Nu V. Vu, David Beran

**Affiliations:** a Foundation Initiatives in Medical Education, Bishkek, Kyrgyzstan; b Division of Tropical and Humanitarian Medicine, Geneva University Hospitals, Geneva, Switzerland; c Unit of Development and Research in Medical Education (UDREM), University Faculty of Medicine, Faculty of Medicine, Geneva, Switzerland; d Swiss Agency for Development and Cooperation, Bishkek, Kyrgyzstan; e Division of Tropical and Humanitarian Medicine, University of Geneva, Faculty of Medicine and Geneva University Hospitals, Geneva, Switzerland

**Keywords:** Education, medical, education, medical, undergraduate, education, medical, graduate, education, medical, continuing, health care reform

## Abstract

Human resources are one of the six building blocks of a health system. In order to ensure that these resources are adequately trained to meet the evolving needs of populations, medical education reforms are needed. In Kyrgyzstan, like in many other low- and middle-income countries, human resources for health are a key challenge for the health system in both the quantity and having their training aligned with the health system priorities. Here we present the experience of the Medical Education Reform Project, a project aimed at improving the quality of health professionals through reforming medical education, funded by the Swiss Agency for Development and Cooperation, as a collaborative effort between partners in Kyrgyzstan and Switzerland since 2013. We used a qualitative study taking a cooperative inquiry approach with an experiential perspective in order to present the implementation of the Medical Education Reform Project in Kyrgyzstan. In order to look at the different components impacting the reform process, a framework comprising: Setting the direction; Building a consensus; Engaging stakeholders; Pilot projects and evaluation; Capacity building; Timing, and Key partners was used to disentangle the lessons learnt. Champions and partnering with key institutions were essential in building consensus, as was the catalytic and facilitating role the project played. This enabled active engagement of a variety of stakeholders in the reform process using different means of interaction ranging from large roundtable discussions, workshops, trainings and even study tours. Pilot projects and research provided tangible actions that could be used to further the reforms. For capacity building, the project offered a wide range of activities that improved clinical competencies, empowered stakeholders, and strengthened organizational capacity. The timing of this reform process in medical education was facilitated by the overall reforms and policies in the health system.

## Background

The Organisation for Economic Co-operation and Development (OECD) defines a reform as a ‘process in which changes are made to the formal “rules of the game” – including laws, regulations and institutions.’ [1] The changes are made to tackle a specific problem or to attain a specific target and involve ‘a complex political process, particularly when it is perceived that the reform redistributes economic, political, or social power.’ [1] There is the need to reform medical education in order to meet the needs of the health system and the population served and link human resource planning with education and training [2–4]. Reforms in medical education need to focus on many related areas such as medical school curricula [5], models of training (e.g. time-based model to competency-based) [6], residency programs [7] or even financing [8,9]. Given changes in the environment, e.g. economic, social and disease trends, reforms are required to adapt the training of health professionals to the new realities they will face in the health system [4]. Frenk et al. [10] have stated that globally medical education has not adapted to the current state of health of populations due to the curricula being fragmented, outdated and unchanged for many years. These factors lead to graduates being poorly equipped to face the challenges they will face as doctors [10,11].

Training for medical professionals entails Pre-graduate Medical Education (PGME), Postgraduate (PME) and Continuing Medical Education (CME) for both doctors and nurses. This medical education training leads to human resources within the health system providing services which are one of the six building blocks of a health system as defined by the World Health Organization (WHO) [12], as well as being an essential component in achieving Universal Health Coverage (UHC) [2].

Kyrgyzstan, located in Central Asia, has been recognized internationally for its improvements in access to care and coverage for its population [13,14]. Despite this success, challenges have been seen in regard to the number [15] and training of health professionals especially at Primary Health Care (PHC) level [16] with this challenge recognized in different health strategies [17–19]. Many of these problems regarding human resources within the health system have their roots in the way medical education functions. For example, financing for medical education is insufficient requiring medical faculties to have too many students for their actual capacity and attract foreign students as a source of income. In 2013 in Kyrgyzstan there were 24.9 medical graduates per 100,000 population and 73.3 nursing graduates per 100,000 in comparison to 11.4 and 40.9 per 100,000 in WHO European Region, respectively. Despite these high numbers of graduates in Kyrgyzstan there were only 63.0 physicians and 558.2 nurses per 100,000 population versus 84.0 and 715.7 in WHO European Region [20]. Staffing of medical doctors shows a major difference between Bishkek (capital city) and the regions, with 100% of positions are filled in Bishkek in comparison to 44% in the regions. In comparison, 97% of nurse positions are filled [14]. In addition, a large migration of health professionals from the country, poor working conditions, an ageing workforce, and low starting salaries US$ 150–200 per month compound the challenge of human resources in Kyrgyzstan [15]. Over the last decade, Kyrgyzstan has embarked on a major healthcare reform, reducing the overall hospital capacity, moving towards more ambulatory care, retraining and developing a stronger primary care base with General Practitioners (GP)/Family Medicine (FM) doctors. In this paper, we present the experience of the Medical Education Reform (MER) Project in reforming medical education and provide key lessons learnt with regard to this process.

## Medical Education Reform Project

Over the last decade, Kyrgyzstan has been actively involved in major health care reforms [17–19]. These systemwide reforms have focused on the strengthening of PHC with the development of GP and FM as key elements with regard to human resources. Given these changes in human resources included in this overall reform of the health system, reforms in the area of medical education are also recognized as essential. These reforms in medical education have been underway since 2007, with financial support provided by the Swiss Agency for Development and Cooperation (SDC) and the technical support from the Geneva University Hospitals (HUG) and University of Geneva Faculty of Medicine (UGFM). As of 2007, the focus of the project was on PGME, concentrating on the design, reorganization and implementation of reforms in this area of medical training. Recognizing the need to extend these reforms beyond only PGME in 2013 two components of PME and CME were added. In addition, at this stage a local implementing partner, Initiatives in Medical Education (IME), joined the HUG and UGFM teams to provide local support and expertise. This combined team formed the Medical Education Reforms (MER) project and has successfully worked together since 2014 to address the different barriers to reforming medical education at these three levels. In 2017, a nursing component was also added to the project.

Overall the main achievements of the project at PGME level have been: the implementation of a new, more integrated curriculum for Years 1–6; introduction, approval and continuous revisions of Kyrgyz State Educational Standards and Undergraduate Learning Objectives and Competencies; Establishment of Kyrgyz State Medical Academy’s (KSMA: main Medical Faculty located in Bishkek) curriculum organization structure and governance; Training KSMA faculty on curriculum design and development and on approaches and techniques in students’ assessment and curriculum evaluation; Introduction of Training of the Trainers courses on curriculum development and revision, and techniques in teaching and in clinical training; Engagement at Osh State Medical University (Medical Faculty located in South of Kyrgyzstan) in the PGME curriculum reform process; and Engagement of both the Ministry of Education and Science and the Ministry of Health in revising aspects of the system, policies and environment to enable medical education reforms and health personnel working capacity.

For PME, a national strategy for the reform has been developed which also includes CME; Development and approval of a new regulation; Pilot project in one region on the decentralization of PME; Involvement of a variety of stakeholders in different aspects of the changes to PME; and shift from the Ministry of Education to the Ministry of Health being responsible for PME. As for CME: Different approaches to delivering CME have been tested; Development and implementation of a comprehensive approach to e-learning; and Creation of the Kyrgyz Medical Association (KMA) and its active involvement in delivering CME. For the nursing component, which is much earlier in its reform process, most of the work has focused on defining the role of nurses in the Kyrgyz context as well as curriculum reforms. All these changes require regulations and legislation to be amended.

## Methods

In order to explore the lessons learnt from the implementation of this project and its impact on the reform process, a qualitative approach was taken using a cooperative inquiry approach as proposed by Heron and Reason [21]. This method includes the participants of the project actually contributing to the research and analysis. In this study, all the individuals involved are responsible for different components of the overall project, thus taking an experiential perspective to this inquiry. The process was iterative in using documents and materials prepared by the project, meetings, minutes from meetings and discussions to analyze the project using the elements of education reform proposed by Schleicher [22]. Schleicher [22] proposes a model for reforms in education including a variety of elements seen as essential in the reform process (Table 1). The last component included in Schleicher’s model is Teacher Unions, which has been replaced by Key partners in reforms for the purpose of this analysis.

**Table 1.** Elements needed for education reform [18]

Element	Definition
Setting the direction	Communicating the overall goal of what will be achieved
Building a consensus	‘Strategic leadership’ Development of consensus through regular interactions with stakeholders Consultations with local partners
Engaging stakeholders in the process of the reforms	Regular interactions Trust building Keeping an open dialogue
Pilot projects and evaluation	Using pilot projects to build consensus Review and evaluate processes
Capacity building	Strengthening capacity
Timing	Importance of the ‘timing’ of the reforms
Key partners	Key institutions and groups of individuals necessary for successful reforms

All authors developed a list of main achievements and lessons from the project. This serves as a basis for the initial analysis. Initial feedback was then provided by some co-authors before all other authors further contributed to its finalization.

## Results

Below, the findings are presented using the elements of education reform proposed by Schleicher [22].

### Setting the direction

The overall directions established by the project team with local stakeholder were as follows for each component of the MER Project: UGME focused on medical students acquiring general clinical competencies before entering PGME; PGME focused on practice-based training away from the capital city; CME training to be integrated into daily practice; and for nursing defining the important role of nurses in healthcare provision. Overall, the main messages at the UGME, PGME and CME were the need for medical education to prepare human resources for their role at PHC level with a focus on FM and GP for doctors.

With regard to setting the direction, four elements are important from the reform perspective. The first relates to a strategic orientation on focusing on FM and PHC. This focus required much work by the project to clearly define what was meant by FM and PHC in the context of Kyrgyzstan as there was much confusion around this term at all levels of medical education, as well as within the medical profession and Ministry of Health. Despite these limitations, the second component, was the clear requests from the Ministry of Health with regard to the need for reforms and meeting the health needs of the population. This was translated into the different national health strategies which included the issue of human resources for health.

Another piece that was essential was that the alignment with the strategy of the SDC, a key member of the Kyrgyz donor community during the period of this project. This enabled the project and its approaches to be promoted within the donor community as well as within the Kyrgyz government.

The final element within ‘Setting the direction’ was the creation of a communication strategy. This was the result of a crisis around the implementation of the new PGME strategy and a misunderstanding within the Kyrgyz context of how to reinforce FM with pushback from students and the medical faculties.

### Building a consensus

Strategic leadership was present for this project at different levels. First, at the level of the Kyrgyz government, certain key individuals were identified and became ‘champions’ for the project. Champions were also present at the medical faculties as well as in the health system. To complement this, SDC leadership and the management of the project enabled a cohesive core group of individuals who were able to work together to foster progress in different areas of the reform. Also, through involving the vice-speaker of the Parliament in different high-level roundtables on the reforms, this gave additional political impetus to this process.

One example from the overall reforms was the need to decentralize PGME to the regions. Key individuals within the Ministry of Health, medical faculties, and health system strongly supported this. Reasons for supporting this varied between different stakeholders, with some focused on the quality of training, versus others on overburdened training centers in the capital city, or even the need for additional human resources in regions. This example is also relevant in looking at developing consensus through interactions with stakeholders. Throughout the project, these interactions took various forms, including: informal meetings; roundtable discussions, workshops, trainings, establishing platforms and working groups, policy dialogues, conferences, and even study tours involving a wide-range of stakeholders, including regional representatives. These different formats allowed for diverse ways of presenting the same issues that the project needed to address. For example, for the UGME reforms, meetings between the project team and key individuals were complemented with specific workshops, e.g. on training the trainers workshops at the oblast levels, on examinations, as well as study visits to Geneva by Kyrgyz colleagues to see how things were done in Geneva as well as have the opportunity to exchange experiences between colleagues.

The project also provided a catalytic and facilitating role in developing consensus between the variety of stakeholders involved in reforming the different components of medical education. This was done, for example, by supporting platforms, e.g. at UGME level, a platform between the Ministries of Health and Education, Ministry of Health and different medical faculties, the creation of the KMA or establishing a coordinating committee to coordinate and monitor all PGME activities.

These different modes of interacting with stakeholders besides allowing for consensus to be developed were also part of ongoing consultations for improving and furthering the reform process. One such area was the development of the nursing component. This required a variety of consultations with local partners from the Ministry of Health, Nursing associations, nursing schools, etc. These consultations required an investment in time to develop and present ideas for the reforms in Kyrgyzstan, different series of interactions with different experts. One element in particular that needed consensus building was the role of nurses in delivering care in Kyrgyzstan. All these exchanges resulted in the adoption in 2018 of a strategy on nursing by the Ministry of Health.

### Engaging stakeholders in the process of the reforms

Core components included having a local team with international support plus the relationship and trust developed over time with other partners. Another key engagement was that of involving regions in a variety of activities. These activities in turn reinforced the relationships and trust with local partners. Both the activities and the relationships and trust are also strengthened by the research component within the project as this helped inform the activities and how they are developed as well as showing local partners the importance the MER Project places on clearly understanding the local context. All these elements allow for policy reforms in a difficult area to be implemented. This in turn contributes to strengthening the relationship and trust of the MER Project with local partners. Also, using the experience from the regions helped influence policy at a central level as well as linking practical experience to changes in policy.

One example of this has been the creation of the KMA. Regular meetings with the different PMAs and their involvement throughout the project, on issues related to PGME and CME resulted in trust being built with the MER project. This trust enabled discussion on the creation of the KMA in both formal roundtables as well as informal meetings. These discussions were complemented with the MER project carrying out a small study with the presentation of this report having a significant impact in bringing together many of the PMAs in Kyrgyzstan for the first time and initiating a discussion on the role of PMAs and how they could work together which eventually resulted in the creation of the KMA.

A clear lesson in engaging local stakeholders was the importance of having organized a study tour to Geneva of key decision makers at the beginning of the development of the UGME, PGME and CME components of the project. Another element that helped build the relationship and trust was the development of certain additional activities in the areas of palliative care, cardiovascular disease rehabilitation, gastroenterology, angiology, pulmonology, rheumatology, renovation of dormitories, provision of computers for e-learning, etc.

### Pilot projects and evaluation

Many of the activities initiated that built trust were clinical activities and also pilot projects which enabled new methods and approaches to be tested. For example, involving nurses in palliative care allowed for a new approach to be developed on a specific health issue as well as highlighting the important role of nurses working with doctors. For UGME, there was a comprehensive and longitudinal new curriculum program evaluation which included the evaluation of students and faculty of the new curriculum and activities.

In addition, these projects enabled consensus to be built on certain key elements of the reforms. A key example of this was the decentralization of PGME, where initially there was much resistance to this concept. To address this the project, for example, funded the rehabilitation of dormitories and through trust built with a variety of regions in Kyrgyzstan was able to push ahead with the decentralization of PGME. Leadership by key regional stakeholders was essential, as was communication, especially to residents about the benefits of leaving the capital city for their training. For this component, different tools were developed to monitor and evaluate different elements of this area of the project, such as a questionnaire administered regularly by supervisors and a personal logbook.

Monitoring and evaluation were also essential, and a series of indicators were developed and routinely collected to assess progress as well as act in changing approaches when necessary. Another lesson learnt was the importance of research in aiding to develop the response and activities of the project. An example is the review carried out on PGME and CME showing that there were no regulatory barriers to decentralization of these elements despite this being stated as a barrier by local partners. In addition, other research projects were carried out providing not only a situation analysis or baseline, but also material to further help develop trust by providing a clear understanding of the local situation.

### Capacity building

The project implemented capacity development at three levels: individual, institutional and systems. At the individual level, this included for UGME training seminars of faculty professors in pedagogy skills development, including lecturing, bedside training and clinical supervision, critical thinking, clinical decision-making approach, telemedicine, etc. For PGME regional clinical supervisors were trained, and CME capacity was developed using e-learning approaches.

For institutions, capacity building focused on the development of internal policies, organizational and procedural restructuring. Exchanges between Kyrgyzstan and Switzerland helped develop capacities in the area of CME. The project helped with the introduction of an electronic system for quality control in evaluating the educational processes and students’ knowledge level, as well as providing equipment for video conferences. A system for distance learning system was established benefiting both PGME and CME. For CME, a whole new ecosystem was created with the development of regulations on the accumulation of credit hours for CME, development of a unified database to register CME activities and credit hours, active involvement of PMAs in developing and delivering material, strengthening e-learning capacity, development of PRGs and including nurses in CME. The creation and support of the KMA was also part of strengthening institutional capacity as well as the overall environment by developing a new stakeholder to fill existing gaps within the system.

Finally, at a systems level, one activity served a dual purpose of increasing knowledge and empowering local stakeholders. This was the organization of a Forum on FM which enabled competencies to be gained through the content and workshops included in this event, as well as empower stakeholders active in the area of FM to bring more attention to their role within the reforms.

### Timing

System-wide factors acted as facilitators for the reform process with regard to timing. Firstly, the epidemiological change occurring in Kyrgyzstan with the main causes of morbidity and mortality being NCDs. Next, the overall process of ongoing changes to the health system following the collapse of the Soviet Union, with many of these focusing on increasing the capacity of the system at PHC as well as on its financing. Human resources for health figure prominently in the current national health strategy, which recognizes three core challenges with regard to human resources for health in Kyrgyzstan: namely capacity, ageing of doctors and migration of human resources to other countries.

### Key partners

A variety of stakeholders needed to be included in the overall reform process, including medical students, residents, practicing health professionals, representatives of different non-governmental organizations, professors, medical faculties, schools of nursing, medical facilities, as well as government representatives and governmental institutions. Within this spiderweb of individuals and organizations in a context such as Kyrgyzstan, many individuals wear multiple hats so that speaking and interacting with one individual might mean taking a different approach based on which institution they are representing. Certain key champions were also essential in moving different components of the reforms forward. Beyond Kyrgyz stakeholders such a project involves other organizations such as the World Health Organization and donors in Kyrgyzstan. Having the SDC as a donor able to assist with the strategic direction of the project and also serve as a link to the donor community and policy-makers was also essential.

The Summary of the lessons learnt are presented in Table 2.

**Table 2.** Summary of lessons learnt

Element	Lesson(s) learnt	Persisting challenges
Setting the direction	System-wide changes in the health system with a focus on FM and PHC; focus on health needs of the population; adapt medical education and capacity building within the environment and culture of Kyrgyzstan as much as possible; strong role of Ministry of Health; role of donor in assisting with setting the direction; development of communication strategy	Strengthen focus on role of Ministry of Education in medical education reforms; link between medical education and health system reforms with Ministry of Health; challenge with the perceptions of roles of doctors and nurses and specialists versus FM; Financing mechanisms of UGME and PGME
Building a consensus	Champions; involving a wide range of stakeholders; fostering interactions; catalytic and facilitating role of the project; many consultations with stakeholders; need for coordinated leadership between Ministry of Health and Ministry of Education	Need to consider financial interests, especially those of medical faculties that might be counter to the reform process, for example their role in PGME. Make institutions self-appropriate the reforms made
Engaging stakeholders in the process of the reforms	Importance of international support; engaging stakeholders in the different regions; development of relationships and trust between a wide range of stakeholders; study tours; additional activities in clinical training to foster engagement and interactions; Knowledge and understanding of context (top down regulations and approaches and cultural sensitivities (e.g. managing hierarchy)	Engaging non-health actors, especially Ministry of Education; coordination at different levels of medical and nursing education as well as health system; Involving the PHC and FM stakeholders in academic processes
Pilot projects and evaluation	Use of pilot projects to show the feasibility of certain changes as well as using this experience to further influence changes in reforms; use of research activities to help inform and direct the process of reforms	Resources are significant for the implementation and adequate documentation of pilot projects to then be used in reform process and further integration into the system
Capacity building	Mix of activities to increase clinical competencies; institutional capacity, including formal trainings, workshops, conferences	Evolving needs as project advances.
Timing	Wide ranging changes in the health system	Alignment has occurred for PGME and CME and to a certain extent nursing, but not for UGME, with the needs of the health system
Key partners	Partners from different organizations (Ministry of Health; Actors beyond the health and education sectors; high-level central government officials; and representatives from the regions and clinical practice)	Regular changes in key positions

## Discussion

Although the model proposed by Schleicher [22] was developed for reforms in education, it provides a useful framework for other reform processes. Overall, the lessons learnt from this project are that from the beginning there needs to be a strategic direction set by multiple actors. Strong leadership is needed supported by a group of experts enabling practical approaches to be developed in parallel to addressing hurdles in the reform process. This leadership needs to be complemented by developing relationships and trust with a wide range of stakeholders, as well as the essential role the SDC plays with both the Kyrgyz authorities and donor community. These relationships and trust are fostered by engaging partners in different ways from one-on-one meetings to large policy roundtables as well as practical activities and pilots. These undertakings were supported by research and monitoring and evaluation activities which strengthen the activities as well as provide some validation of approaches and other opportunities to engage partners. These elements together allow for the reform process to advance.

**Figure 1.**
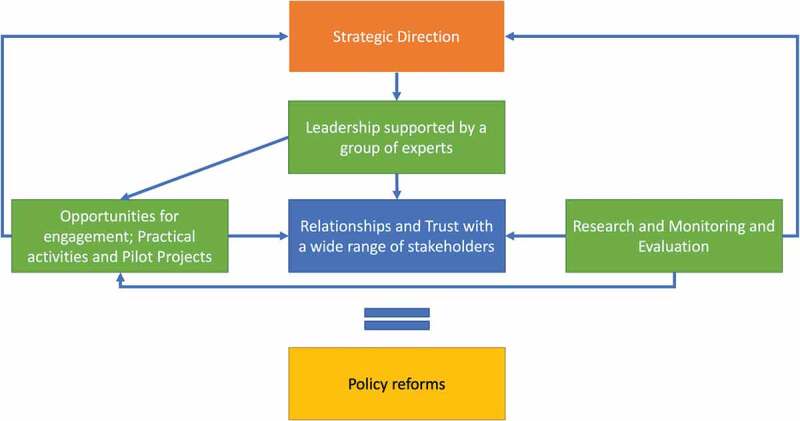
Summary of key components of reform process

Although this experience focuses on medical education, the lessons learnt from this project on reforms in health and the approaches and tools needed will be of interest for other projects trying to change complex systems. The approach developed by the MER project resulted in empowering local stakeholders by equipping them with the necessary tools and resources for change and putting them at the center of the reform process (Figure 1). The strategic direction was set by local stakeholders in collaboration with the project and then supported by the SDC. In green are what a project can tangibly bring to the reform processes such as expertise, engagement opportunities, pilot projects, monitoring and evaluation and research. By using these different approaches and tools the intangible element of relationships and trust is created. This element of trust and relationships has been highlighted as essential in fostering partnerships for successful projects [23,24]. By continuously engaging stakeholders as well as using research and monitoring and evaluation also help adapt the strategic direction of the reforms accordingly.

Human resources are a key element of health systems. For medical education, it is not just one organization or institution that is required to change, but multiple pieces of the overall delivery of medical and education and health system. As stated by Braithwaite et al. [25] the reform process requires changes in ‘politics, cultural shifts, the mobilization of power and the exercise of resource reallocations’ as well as being intimately linked to the country’s ‘economy, culture, geography, socio-economic circumstances, population size and its political frame-work and relative stability or instability.’

## Conclusions

Any reform process is complex. Reforms in medical education cannot be made in isolation of reforms in the health system in addition to changes being needed in organizations, their governance and roles. This results in diverging interests which include the competing agendas between medical education reform and health systems reform, as well as the need to involve the Ministry of Education in reforms that go beyond their mandate of university training and impact the delivery of health. The lessons from this project show that championing and partnering with key institutions were essential in building a consensus, as was the catalytic and facilitating role the project played. This enabled active engagement of a variety of stakeholders in the reform process using different means of interaction ranging from large roundtable discussions, workshops, trainings and even study tours. Pilot projects and research provided tangible actions that could be used to further the reforms. For capacity building, the project offered a wide range of activities that improved clinical competencies, empowered stakeholders, and strengthened organizational capacity. The timing of this reform process in medical education was facilitated by the overall reforms and policies in the health system.

## Data Availability

All reports and material are available at http://www.ime.org.kg/ or on request from the authors.

## Paper context

Education reforms are needed to align health worker competencies with population health needs. We present the Medical Education Reform project in Kyrgyzstan, which since 2013 has worked to improve the quality of health professionals through reforming medical education. The lessons from this project in order to implement successful reforms are the: catalytic effect and facilitating role of external support; importance of working with champions; and partnering with key institutions to build a consensus.
